# Drugs-Related Death Soon after Hospital-Discharge among Drug Treatment Clients in Scotland: Record Linkage, Validation, and Investigation of Risk-Factors

**DOI:** 10.1371/journal.pone.0141073

**Published:** 2015-11-05

**Authors:** Simon R. White, Sheila M. Bird, Elizabeth L. C. Merrall, Sharon J. Hutchinson

**Affiliations:** 1 Medical Research Council Biostatistics Unit, Cambridge Institute of Public Health, Cambridge, United Kingdom; 2 Novartis Pharam BV, Novartis Vaccines & Diagnostics, Hullenbergweg 83–85, 1101 CL Amsterdam, Netherlands; 3 Health Protection Scotland, Glasgow, G3 7LN, Scotland, United Kingdom; Penn State College of Medicine, UNITED STATES

## Abstract

We validate that the 28 days after hospital-discharge are high-risk for drugs-related death (DRD) among drug users in Scotland and investigate key risk-factors for DRDs soon after hospital-discharge. Using data from an anonymous linkage of hospitalisation and death records to the Scottish Drugs Misuse Database (SDMD), including over 98,000 individuals registered for drug treatment during 1 April 1996 to 31 March 2010 with 705,538 person-years, 173,107 hospital-stays, and 2,523 DRDs. Time-at-risk of DRD was categorised as: during hospitalization, within 28 days, 29–90 days, 91 days–1 year, >1 year since most recent hospital discharge versus ‘never admitted’. Factors of interest were: having ever injected, misuse of alcohol, length of hospital-stay (0–1 versus 2+ days), and main discharge-diagnosis. We confirm SDMD clients’ high DRD-rate soon after hospital-discharge in 2006–2010. DRD-rate in the 28 days after hospital-discharge did not vary by length of hospital-stay but was significantly higher for clients who had ever-injected versus otherwise. Three leading discharge-diagnoses accounted for only 150/290 DRDs in the 28 days after hospital-discharge, but ever-injectors for 222/290. Hospital-discharge remains a period of increased DRD-vulnerability in 2006–2010, as in 1996–2006, especially for those with a history of injecting.

## Introduction

Injecting drug users experience significantly higher mortality rates [1–13], and identifying opportunities of intervention, such as upon release from prison or expiry of methadone prescription [5, 10], are important for public health policy.

Scotland has invested in record linkage for drug users to monitor blood born viruses and prevalence of injecting drug users. Using record linkage, Merrall et al. [14] added to the literature of drug user mortality by showing that drug treatment clients were at increased risk of drugs-related death (DRD) within 28 days after hospital-discharge in Scotland in 1996–2006 (21 DRDs per 1,000 person-years (pys); 95% CI: 18, 25).

In this article, we first validate the findings by Merrall et al. [14] for 1996–2006 by investigating SDMD clients’ DRD-risk by time after hospital-discharge in 2006–2010. The injector population is changing over time, as the progression and initiation of injectors changes through social change and public health interventions, so it is important to validate past findings. Secondly, using the entire 1996–2010 SDMD cohort with the increased person-years of follow-up–and hence increased statistical power- we are able to investigate more detailed hospital episode characteristics, such as duration of hospitalization and main discharge-diagnosis.

We confirm SDMD clients continue to experience higher drugs-related mortality rates after hospital-discharge, and further that having been an injection drug user better identifies those at highest DRD-risk soon after hospital-discharge than duration of hospital-stay or main discharge-diagnosis.

## Methods

The study of hard-to-reach populations, such as people who inject drugs, has been hugely facilitated by linkage between administrative records and confidential health registers, see for example [3, 4, 6, 7, 9–13]. Scotland’s Information Services Division holds the Scottish Drugs Misuse Database (SDMD), which records all registrations in Scotland for drug treatment or support. By a variation on the Privacy Advisory Committee permissions for Scotland’s surveillance of the late sequelae after hepatitis C virus diagnosis [15], we could access linked data for the present study of SDMD clients’ DRDs to 31 March 2010.

### Study population and data sources

For each drug treatment registration in Scotland, the SDMD holds limited identifying information: sex, date of birth, forename initial, first and fourth letter of the surname, and postcode sector of residence. Data are also held on risk behaviors such as illicit drugs used, reported misuse of alcohol, and injecting drug user status at the time of SDMD registration. Linked SDMD records were available on 98,388 individuals who attended drug treatment services between 1 April 1996 and 31 March 2010.

Deaths, hospital episodes and hepatitis C virus diagnoses of individuals registered on the SDMD during April 1996 through March 2010 were identifiable through linkages with the national registers held respectively by Information Services Division, National Records of Scotland (formerly the General Register Office for Scotland) and Health Protection Scotland. Records were linked by Information Services Division using a probabilistic approach on the available identifying information at the time of linkage. Note that these identifiers may have changed between the linkages performed in 2006 and 2010. For each SDMD client, potentially corresponding death, hospital records and hepatitis C virus records were ranked according to a linkage score which was based on a probabilistically-weighted combination of the occurring identifiers. The top-ranked match would be successful if the score exceeded a pre-determined threshold (see Merrall [16] for further details).

The linked dataset was anonymized before transfer to Medical Research Council Biostatistics Unit for analysis. This anonymization, together with updating of contributory records, explains why linked-records for the same individual cannot assuredly be matched between successive linkages, such as for the 1996–2006 and updated 1996–2010 SDMD cohorts. Information Services Division has previously estimated its procedure to have an error rate (either false positives or false negatives) of less than 5% [17]. However, low-level inconsistencies inevitably remain. Moreover, due to the updating of the contributory linkable records, the SDMD cohort for 1996–2006 is now reckoned as 74,654 registered clients (previously 69,457 [14]). There are thus actual differences in the client, DRD, and person-year counts from those reported by Merrall et al. [14] for 1996–2006 but these differences have had no implications for the inferences drawn, which are robust.

### Statistical analysis

We investigated DRDs as nationally defined by Jackson [18] and National Records of Scotland, which comprise deaths involving drugs or attributed to one’s drug dependence and have the following groupings: mental and behavioral disorders due to psychoactive substance misuse; accidental poisoning; intentional self-poisoning by drugs, medicaments and biological substances; assault by drugs, medicaments and biological substances; and events of undetermined intent, poisoning. See Table 1 for the specific International Classification of Disease codes (9^th^ & 10^th^ version) corresponding to each grouping.

**Table 1:** ICD codes for cause of death or hospital discharge.

Classification	ICD 9	ICD 10
Infectious and parasitic diseases	001–139	A00–B99
Cancer	140–208	C00–C97
Endocrine, nutritional and metabolic diseases	240–279	E00–E89
Mental and behavioural disorders	290–319, Excluding: 305.2–305.9	F00–F99, Excluding: F11–F16, F19
Diseases of the nervous system	320–389	G00–G99
Diseases of the circulatory system	390–459	I00–I99
Diseases of the respiratory system	460–519	J00–J99
Diseases of the digestive system	520–579	K00–K93
Accidental	E800–E949, Excluding: E850–E858	V01–X59
(non-DRD) Suicide	E950.6–E959	X65–X84
Homicide	E960–E969, Excluding: E962.0	X86–Y09
Drugs-related death^a^		
Mental and behavioural disorders due to psychoactive substance misuse	292, 304, Excluding: 304.6	F11–F16, F19
Accidental poisoning	E850–E858	X40–X44
Intentional self-poisoning by drugs, medicaments and biological substances	E950.0–E950.5	X60–X64
Assault by drugs, medicaments and biological substances	E962.0	X85
Events of undetermined intent, poisoning	E980.0–E980.5	Y10–Y14
Drugs-related morbidity^b^		
Poisoning by drugs, medicaments and biological substances	960–976	T36–T50
Mental and behavioural disorders due to psychoactive substance misuse	292, 304 excluding 304.6	F11–F16, F19

Abreviations: DRD, drugs-related death; ICD, International Classification of Disease

^a^ Any codes corresponding to drugs-related causes were excluded from other categories, so all are mutually exclusive

^b^ Category used only for hospital discharge diagnoses

Each hospital record corresponds to an episode of care within a general/acute or mental health specialty, respectively, in Scotland. The record is generated when a patient is discharged or transferred between hospitals, specialties or consultants [19], and the duration of an episode may range from a day-visit up to a stay of several months. Hence, a single hospital-stay may comprise a series of episodes. Therefore, for each SDMD client, serial hospital episodes with overlapping or matching end- and start-dates were coalesced as a single hospital-stay which began at the set’s earliest start-date and ended on the latest end-date.

Duration of hospital-stay was computed as ‘latest end-date minus earliest start-date’, and is thus measured in whole days with respect to the recorded admission and discharge dates. The majority of hospital-stays were of one day or less, a large number were computed as zero-length. Within the data it is not possible to derive an exact admission time, so we cannot distinguish between a short-stay that occurs over night and a stay for at least one day. Accordingly, duration was summarized as 0–1 days versus 2+ days, the former accounting for brief episodes that may or may not have included an over-night stay. The length of hospital-stay is highly skewed, and exhibits a so-called zero-inflated distribution (meaning there are many individuals with zero-length stays). With insufficient individuals to properly characterize longer stays, we dichotomize into (conservatively defined) day visits (0-1days) or longer (2+ days).

Hospital episodes are recorded with a main discharge-diagnosis and supplementary discharge diagnoses [4, 16]. We restrict attention to the main discharge-diagnosis of the last episode for each hospital-stay. The discharge codes for drug-related morbidity were classified using the same groupings as for drug-related mortality but with the addition of poisoning by drugs, medicaments and biological substances and mental and behavioral disorders due to psychoactive substance misuse. Further major categories of discharge-diagnosis were: infectious and parasitic diseases; cancer; endocrine, nutritional and metabolic diseases; mental and behavioral disorders—excluding those due to psychoactive substance misuse; diseases of the nervous system; diseases of the circulatory system; diseases of the respiratory system; diseases of the digestive system; and accidental. See Table 1 for the specific International Classification of Disease codes (9^th^ & 10^th^ version) corresponding to each grouping.

For the validation analysis, time-at-risk was from 1 April 2006 or the date of an individual’s first attendance at drug treatment services if after 1 April 2006, until the earlier of date of death or end-of-study, 31 March 2010. Time since most recent hospitalization was categorized as: during hospitalization, within 28 days, 29–90 days, 91 days–1 year, >1 year after discharge from most recent hospital-stay *versus* ‘never admitted’ (reference category). To be conservative, deaths which occurred on the end-date of a hospitalization were counted as hospitalized deaths. As in Merrall et al [14], hazard ratios from Cox proportional hazards analysis [20], with adjustment for time-dependent covariates, are also reported.

For adequately-powered secondary analyses of behavioral risks (ever injecting drug users; misuse of alcohol) and hospitalization covariates (length of stay; main discharge-diagnosis), we considered the 1996–2010 SDMD cohort in its entirety so that at least 30 DRDs in the 28 days after hospital-discharge might be available for analysis per discharge-diagnosis. Time-at-risk in secondary analyses was from the date of an individual’s first attendance at drug treatment services after 1 April 1996 until the earlier of date of death or end-of-study, 31 March 2010.

All statistical analyses were conducted using R version 2.15.0 [21].

## Results

### Characteristics of study population

Table 2 presents the characteristics of the study cohort, firstly for the SDMD clients first observed during the era originally studied, April 1996 to March 2006, and secondly for those first observed during April 2006 to March 2010. In the later era, an additional 23,734 individuals were newly registered in the SDMD cohort, with 14,474 subsequent hospital-stays and 168 DRDs during 50,453 person-years of follow-up. At their first registration in the 2006–2010 era, 68% were under 35 years of age (16,057) compared with 83% of those registered in the earlier era; 36% (8,605) had a recorded history of injection drug use (past or present) compared with 49% of those in 1996–2006.

**Table 2:** Descriptive Statistics for characteristics at First SDMD Registration by Registration Era, SDMD Cohort, Scotland, 1996–2010.

Registration era:	Apr 1996–Mar 2006		Apr 2006–Mar 2010	
New SDMD clients (i.e. number of first registrations)	74,654		23,734	
Male	51,856	69.5%	16,931	71.3%
Female	22,798	30.5%	6,803	28.7%
< 25 years	31,326	42.0%	7,408	31.2%
25–34 years	30,861	41.3%	8,649	36.4%
**> 35 years**	**12,467**	**16.7%**	**7,677**	**32.3%**
Injected in past month	24,402	32.7%	4,536	19.1%
Injected, but not in past month	12,485	16.7%	4,069	17.1%
**Never injected**	**33,533**	**44.9%**	**13,776**	**58.0%**
Unknown	4,234	5.7%	1,353	5.7%

Abbreviations: SDMD, Scottish Drugs Misuse Database

Table 3 presents descriptive statistics by follow-up era, with the 98,388 individuals contributing 334,421 person-years in the second follow-up era, 2006–2010, when there were 51,504 SDMD registrations, 78,658 hospital stays (based on 83,084 hospital-episodes and 11,818 psychiatric-episodes) and 2,544 deaths (including 1,114 DRDs). Across eras, DRDs account for about half of all deaths: 1409/2585 (55%) in 1996–2006 but 1114/2544 (44%) in 2006–2010. Non-drug-related suicide accounted for 11% (284) of deaths in the first era, but for only 6% (165) in the second. Conversely, diseases of the digestive system accounted for 6% (165) of deaths in the first era, but for 13% (341) in the second.

**Table 3:** Descriptive Statistics for outcomes by Follow-up Era, SDMD Cohort, Scotland, 1996–2010.

Follow-up era:	Apr 1996–Mar 2006		Apr 2006–Mar 2010	
Total number of records	260,794		149,927	
SDMD		138,020		51,504
Hospital		96,465		83,084
Psychiatric		17,355		11,818
Hepatitis C virus diagnosis		8,954		3,521
Total Deaths	2585		2544	
**Drugs-related deaths**	**1,409**	**54.5%**	**1,114**	**43.8%**
**Non-DRD suicide** ^a^	**284**	**11.0%**	**165**	**6.5%**
**Diseases of the digestive system**	**165**	**6.4%**	**341**	**13.4%**
**Diseases of the circulatory system**	**125**	**4.8%**	**193**	**7.6%**
**Homicide**	**102**	**3.9%**	60	2.4%
Accidental	93	3.6%	82	3.2%
Infectious and parasitic diseases	91	3.5%	65	2.6%
**Cancer**	69	2.7%	**130**	**5.1%**
**Diseases of the respiratory system**	64	2.5%	**103**	**4.0%**
Mental and behavioral disorders	60	2.3%	77	3.0%
Endocrine, nutritional and metabolic diseases	22	0.9%	25	1.0%
Diseases of the nervous system	16	0.6%	21	0.8%
Total hospital-stays	94,449		78,658	(14,474)^b^
Person-years follow-up	371,117		334,421	(50,453)^b^

Abbreviations: DRD, drugs-related death; SDMD, Scottish Drugs Misuse Database

^a^ Excluding DRD suicides which are included in the DRD total,

^b^ Restricted to individuals with first SDMD registration in 2006–2010 as in Table 1(a)

The overall DRD-rate was 3.8 DRDs (95% CI: 3.6, 4.0) per 1,000 person-years in 1996–2006 but reduced to 3.3 DRDs (95% CI: 3.1, 3.5) per 1,000 person-years in 2006–2010.

#### Validation analysis: drugs-related deaths by time since hospitalization

Table 4 summarizes DRD-rates by time since hospitalization in 1996–2006 and in the validation era of 2006–2010. In both eras, DRD-risk was highest during hospitalization but hospitalized DRD-rate decreased significantly from 74 DRDs per 1,000 person-years (95% CI: 61, 88) in 1996–2006 to 50 (95% CI: 39, 63) in 2006–2010. After discharge from a hospital-stay, DRD-rates per 1,000 person-years were consistent between eras: 24.6 and 22.5 within 28 days; 12.0 and 12.0 during 29–90 days; 8.3 and 8.6 for the remainder of the first year.

**Table 4:** Drugs-related Death Rates by Follow-up Era and Time Since Hospital-discharge: Unadjusted, SDMD Cohort, Scotland, 1996–2010.

By follow-up era:	Apr 1996–Mar 2006				Apr 2006–Mar 2010			
	DRD	Pys	Rate	(95% CI)	DRD	Pys	Rate	(95% CI)
Time since hospital-discharge								
Never Admitted	458	257,643	**1.8**	(1.6, 2.0)	253	209,031	**1.2**	(1.1, 1.4)
Hospitalized	125	1,697	**73.6**	(61.3, 87.8)	75	1,497	**50.1**	(39.4, 62.8)
Within 28 days	165	6,714	**24.6**	(21.0, 28.6)	125	5,556	**22.5**	(18.7, 26.8)
29–90 days	131	10,918	**12.0**	(10.0, 14.2)	107	8,903	**12.0**	(9.9, 14.5)
91 days– 1-year	271	32,698	**8.3**	(7.3, 9.3)	235	27,306	**8.6**	(7.5, 9.8)
1+ years	259	61,446	**4.2**	(3.7, 4.8)	319	82,128	**3.9**	(3.5, 4.3)

Abbreviations: DRD, drugs-related death; SDMD, Scottish Drugs Misuse Database; Pys, Person-years; CI, Confidence Interval

For each follow-up era Table 5 presents Cox Hazard Ratios by time since most recent hospitalization after adjustment for other DRD risk factors. In particular, note the influence of reported misuse of alcohol in addition to having ever injected.

**Table 5:** Drugs-related Death Rates by Follow-up Era and Time Since Hospital-discharge: Adjusted Using Cox Proportional Hazards Regression With Calendar Time as the Underlying Time-scale, SDMD Cohort, Scotland, 1996–2010.

Follow-up era	Apr 1996–Mar 2006		Apr 2006–Mar 2010	
	Hazard ratio (95% CI)		Hazard ratio (95% CI)	
**Age**				
< = 34 years (referent)				
35+ years	**1.16**	(1.03, 1.31)	**1.12**	(1.00, 1.27)
**Sex**				
Female	**0.54**	(0.47, 0.62)	**0.58**	(0.50, 0.68)
Male (referent)				
**Injector status**				
Inject in past month	**1.25**	(1.10, 1.42)	**1.26**	(1.09, 1.45)
Ever injected (referent)				
Never injected	**0.56**	(0.48, 0.66)	**0.64**	(0.54, 0.76)
Unknown	**0.62**	(0.44, 0.87)	**0.61**	(0.42, 0.88)
**Time since last SDMD registration**				
<12 weeks	**1.34**	(1.12, 1.60)	**1.64**	(1.29, 2.10)
3–12 months	**1.09**	(0.94, 1.26)	**1.24**	(1.02, 1.51)
1–2 years (referent)				
2–5 years	**0.80**	(0.69, 0.92)	**0.89**	(0.75, 1.06)
5+ years	**0.44**	(0.34, 0.57)	**0.61**	(0.50, 0.74)
**Misuse sedatives**				
Yes	**1.08**	(0.97, 1.21)	**1.28**	(1.13, 1.46)
No (referent)				
**Misuse stimulants**				
Yes	**0.97**	(0.83, 1.13)	**1.04**	(0.89, 1.22)
No (referent)				
**Misuse cannabis/tobacco**				
Yes	**0.87**	(0.76, 0.98)	**0.80**	(0.69, 0.93)
No (referent)				
**Misuse alcohol**				
Yes	**1.48**	(1.26, 1.73)	**1.45**	(1.26, 1.67)
No (baseline)				
**HCV diagnosis**				
Yes	**1.31**	(1.15, 1.50)	**1.28**	(1.10, 1.48)
No (referent)				
**Time since last hospitalised**				
Hospitalised	**35.47**	(29.01, 43.35)	**32.92**	(25.33, 42.79)
within 28 days	**11.83**	(9.87, 14.18)	**15.09**	(12.12, 18.80)
29–90 days	**5.73**	(4.71, 6.98)	**8.01**	(6.36, 10.08)
91 days– 1 year	**4.15**	(3.56, 4.84)	**5.90**	(4.91, 7.08)
1+ years	**2.44**	(2.09, 2.86)	**2.88**	(2.43, 3.41)
Never admitted (referent)				

Abbreviations: SDMD, Scottish Drugs Misuse Database; CI, Confidence Interval

Consistently between 1996–2006 and 2006–2010 the hazard ratio is increased for reported misuse of alcohol (1.48 and 1.45) and for hepatitis C virus diagnosis (1.31 and 1.28); but reduced for females (0.54 and 0.58), and never-injectors (0.56 and 0.64). Relative to having never been admitted, adjusted hazard ratios by recency of hospital-stay were: 11.8 and 15.1 within 28 days; 5.7 and 8.0 during 29–90 days; and 4.2 and 5.9 for the remainder of the first year.

#### Behavioral risk-factors versus duration of hospital-stay or main discharge-diagnosis

For the entire follow-up period of 1996–2010, Table 6 shows the DRD-rates by time since most recent hospital-discharge, according to SDMD clients’ time-dependent ever injecting drug user status and Table 7 shows the DRD-rates by reported alcohol misuse.

**Table 6:** Drugs-related Death Rates Soon After Hospital-discharge for Ever-IDU Behavioral Risk-factor, SDMD Cohort, Scotland, 1996–2010.

Injector-status:	Ever Injected				Never known to have injected			
	DRD	PYs	Rate	(95% CI)	DRD	PYs	Rate	(95% CI)
Never Admitted	512	250,555	**2.0**	(1.9, 2.2)	199	216,119	**0.9**	(0.8, 1.1)
Hospitalised	156	1,771	**88.1**	(74.8,103.0)	44	1,423	**30.9**	(22.5, 41.5)
Within 28 days	222	6,998	**31.7**	(27.7, 36.2)	68	5,271	**12.9**	(10.0, 16.4)
29–90 days	171	11,506	**14.9**	(12.7, 17.3)	67	8,316	**8.1**	(6.2, 10.2)
91 days– 1 year	380	35,820	**10.6**	(9.6, 11.7)	126	24,183	**5.2**	(4.3, 6.2)
1+ years	458	92,362	**5.0**	(4.5, 5.4)	120	51,212	**2.3**	(1.9, 2.8)

**Table 7:** Drugs-related Death Rates Soon After Hospital-discharge for Reported Misuse of Alcohol Behavioral Risk-factor, SDMD Cohort, Scotland, 1996–2010.

Alcohol misuse:	Yes				No			
	DRD	PYs	Rate	(95% CI)	DRD	PYs	Rate	(95% CI)
Never Admitted	132	60,437	**2.2**	(1.8, 2.6)	579	406,237	**1.4**	(1.3, 1.5)
Hospitalised	41	653	**62.8**	(45.1, 85.2)	159	2,542	**62.6**	(53.2, 73.1)
Within 28 days	89	2,655	**33.5**	(26.9, 41.3)	201	9,615	**20.9**	(18.1, 24.0)
29–90 days	47	4,093	**11.5**	(8.4, 15.3)	191	15,728	**12.1**	(10.5, 14.0)
91 days– 1 year	113	11,324	**10.0**	(8.2, 12.0)	393	48,680	**8.1**	(7.3, 8.9)
1+ years	105	20,493	**5.1**	(4.2, 6.2)	473	123,082	**3.8**	(3.5, 4.2)

Abbreviations: DRD, drugs-related death; SDMD, Scottish Drugs Misuse Database; Pys, Person-years; CI, Confidence Interval; IDU, Injecting Drug User

The DRD-rate during hospitalization is nearly three times higher for ever versus never injecting drug users (88 versus 31 per 1,000 person-years). Moreover, within 28 days of hospital-discharge ever injecting drug users experienced a DRD-rate of 32 per 1,000 person-years (95% CI: 27.7, 36.2) compared with 13 per 1,000 person-years (95% CI: 10.0, 16.4) for drug treatment clients who had never injected. Reported misuse of alcohol, although associated with as high a DRD-rate in the 28 days after hospital-discharge, was a less prevalent risk-factor and did not differentiate DRD-rate during hospitalization.

Table 8 shows DRD-rates within 28 days (and 90 days) after hospital-discharge by each of the following: ever injecting drug use and reported alcohol misuse (from Tables 6 and 7); duration of hospital-stay and main discharge-diagnosis. There is no evident difference in DRD-rate according to duration of hospital stay (0–1 days versus 2+ days). However, we observe some variation in the DRD-rate by discharge-diagnosis.

**Table 8:** Drugs-related Death Rates Soon After Hospital-discharge Within 28 and 90 Days After Hospital-discharge by Duration of Hospital-stay and Main Discharge-diagnosis Versus Ever-IDU and Reported Misuse of Alcohol, SDMD Cohort, Scotland, 1996–2010.

Soon after hospital-discharge	Within 28 days after discharge				Within 90 days after discharge			
	DRD	Pys	Rate	(95% CI)	DRD	Pys	Rate	(95% CI)
Rate for SDMD cohort, 1996–2010	290	12,270	23.6	(21.0, 26.5)	528	32,091	16.5	(15.1, 17.9)
**By injector status**								
Ever-IDU	**222**	6,998	**31.7**	(27.7, 36.2)	**393**	18,504	**21.2**	(19.1, 23.3)
Never or not known to have injected	68	5,271	**12.9**	(10.0, 16.4)	135	13,587	**9.9**	(8.3, 11.6)
**By reported misuse of alcohol**								
Yes	**89**	2,655	**33.5**	(26.9, 41.3)	**136**	6,748	**20.2**	(16.8, 23.5)
No	201	9,615	**20.9**	(18.1, 24.0)	392	25,343	**15.5**	(13.9, 17.0)
**By duration of hospital-stay**								
0–1 day	160	6,781	**23.6**	(20.1, 27.5)	277	17,957	**15.4**	(13.7, 17.4)
2+ days	130	5,488	**23.7**	(19.8, 28.1)	251	14,134	**17.8**	(15.6, 20.1)
**By main discharge-diagnosis**								
Other diagnoses^a^	93	5,836	**15.9**	(12.9, 19.5)	205	16,607	**13.1**	(11.4, 15.1)
Drugs-related morbidity^b^	**74**	1,899	**39.0**	(30.1, 47.8)	**137**	4,996	**27.4**	(22.8, 32.0)
Mental and behavioral disorders^c^	**54**	1,503	**35.9**	(27.0, 46.9)	**80**	3,782	**21.2**	(16.8, 26.3)
Diseases of the respiratory system	22	596	**36.9**	(23.1, 55.9)	34	1,559	**21.8**	(15.1, 30.5)
Cancer	3	116	**25.8**	(5.3, 75.4)	3	199	**15.1**	(3.1, 44.1)
Infectious and parasitic diseases	6	260	**23.1**	(8.5, 50.2)	7	688	**10.2**	(4.1, 21.0)
Diseases of the circulatory system	12	576	**20.8**	(10.8, 36.4)	21	1,502	**14.0**	(8.7, 21.4)
Endocrine, nutritional and metabolic diseases	3	149	**20.1**	(4.2, 58.9)	5	344	**14.5**	(4.7, 33.9)
Diseases of the digestive system	22	1,165	**18.9**	(11.8, 28.6)	32	1,974	**10.8**	(7.4, 15.2)
Diseases of the nervous system	1	170	**5.9**	(0.1, 32.8)	4	440	**9.1**	(2.5, 23.3)

Abbreviations: DRD, drugs-related death; SDMD, Scottish Drugs Misuse Database; Pys, Person-years; CI, Confidence Interval; IDU, Injecting Drug User

^a^ All diagnoses not included in specified groups

^b^ Diagnoses related to drug misuse, (a) Poisonings by drugs, medicaments & biological substances and (b) Mental & behavioral disorders due to psychoactive substance

^c^ All mental and behavioral disorders, excluding those due to psychoactive substances

Among discharge-diagnoses, only two—drug-related morbidity (for poisoning by drugs, medicaments and biological substances plus mental and behavioral disorders due to psychoactive substance misuse) and mental and behavioral disorders excluding those due to psychoactive substances—were associated with at least 30 DRDs within 28 days after discharge and also had high DRD-rates per 1,000 person-years, 39 (95% CI: 30.1, 47.8) and 36 (95% CI: 27.0, 46.9) respectively, as did diseases of the respiratory system, 37 (95% CI: 23.1, 55.9). Together, these top three discharge-diagnoses accounted for only 52% (150/290) of the SDMD cohort’s DRDs within 28 days after hospital-discharge.

By contrast, ever injecting drug users accounted for 77% (222/290) of DRDs in the 28 days after hospital-discharge. The SDMD clients who reported misuse of alcohol had a correspondingly high DRD-rate soon after hospital-discharge but accounted for only 31% (89/290) of all SDMD clients’ DRDs in the 28 days after hospital-discharge.

Ever injecting drug users’ short-term risk can be summarized as one DRD (95% CI: 0.85, 1.11) in the 28 days after hospital-discharge per 400 discharged SDMD clients who had ever injected.

## Discussion

Our key confirmatory finding is that DRD-rates by time since most recent hospitalization remained significantly higher in the 28 days after hospital-discharge than at subsequent times post-discharge (with and without covariate adjustment). The SDMD cohort’s DRD-rate while hospitalized had decreased in 2006–2010, but absolute DRD-risks soon after hospital-discharge remained similar across both periods.

New SDMD registrations in the validation period of 2006–2010 were different from those in the earlier registration period in important respects: a higher proportion of 2006–2010 registered clients were 35 years of age or older at registration than among clients whose first SDMD registration was in 1996–2006, and a higher proportion reported never having injected. This suggests that Scottish drug users are not only ageing but that newer clients are less likely to have been initiated into injecting.

The study disputed our hypothesis that individuals who are hospitalized for a longer time (at least overnight) may be at greater DRD-risk post-discharge due to loss of tolerance. However, the duration of hospital-stay was a highly skewed variable; with a few individuals experiencing very long (several months) periods. There was insufficient statistical power to properly investigate the effect of short versus long stay lengths. A further subdivision of duration of hospital-stay as 0–1 day, 2–6 days and 7+ days (results not shown) also showed no difference between stays of less than versus greater than one week.

The influence of main discharge-diagnosis on subsequent DRD-risk was also analyzed by grouping the codes into pre-specified categories as used previously by Merrall et al. [4]. We needed to consider the entire SDMD cohort in order to have sufficient power per discharge-category and, even so, only two main discharge-diagnoses exceeded 30 DRDs within 28 days after hospital-discharge. These two discharge-categories were drug-related morbidity and mental and behavioral disorders excluding psychoactive substance misuse. Even together with diseases of the respiratory system, these top three DRD-risk discharge-categories accounted for only 52% (150/290) DRDs in the 28 days after hospital-discharge.

By contrast, behavioral risk-factors were far more discriminatory with ever injecting drug use accounting for the vast majority (77%: 222/290) of DRDs in the 28 days after discharge. This finding gives added focus to Scotland’s public health policy to make take-home naloxone (opiate antagonist) readily available, as well as training in its administration, not only in prisons and at drug treatment agencies but also at discharge from hospital, see [22] and [23]. Moreover, our new results suggest how hospital doctors can best target their harm reduction response [24]–not ineffectually according to patients’ length of hospital-stay, nor too narrowly by concentrating on a few main discharge-diagnoses, but highly efficiently by focusing on those who have ever injected. For ever injecting drug users, we note that one DRD in the 28 days after hospital-discharge per 400 discharges is about half their estimated DRD-risk in the 28 days after prison-release [25].

For the SDMD cohort of over 98,000 drug treatment clients in Scotland, we have confirmed that a high DRD risk soon after hospital-discharge applies in 2006–2010 as it did in 1996–2006 [14]. Length of hospital-stay had no effect on DRD-rate, discharge-diagnosis had an effect (as did reported misuse of alcohol) but neither was as discriminatory as the behavioral risk-factor of having ever injected.

## References

[1] Degenhardt L, Bucello C, Mathers B, Briegleb C, Ali H, Hickman M, et al Mortality among regular or dependent users of heroin and other opioids: a systematic review and meta-analysis of cohort studies. Addiction 2010; 106: 32–51. 21054613 10.1111/j.1360-0443.2010.03140.x

[2] SM Bird on behalf of European COSMO Workshop. Over 1200 drugs-related deaths and 190,000 opiate-user-years of follow-up: relative risks by sex and age-group. Addiction Research and Theory 2010: 18 (2): 194–207.

[3] Merrall ELC, Bird SM, Hutchinson SJ. Mortality of those who attended drug services in Scotland, 1996–2006: record-linkage study. International Journal of Drug Policy 2012; 23: 24–32. 21719267 10.1016/j.drugpo.2011.05.010 PMC3271367

[4] Merrall ELC, Bird SM, Hutchinson SJ. A record linkage study of hospital episodes for drug treatment clients in Scotland, 1996–2006. Addiction Research and Theory 2013; 21: 52–61. 10.1111/j.1360-0443.2012.04066.x 22925008

[5] Merrall ELC, Kariminia A, Binswanger IA, Hobbs M, Farrell M, Marsden J, et al Meta-analysis of drug-related deaths soon after release from prison. Addiction 2010; 105 (9): 1545–1554. 20579009 10.1111/j.1360-0443.2010.02990.x PMC2955973

[6] Kariminia A, Butler TG, Corben SP, Levy MH, Grant L, Kaldor JM, et al Extreme cause-specific mortality in a cohort of adult prisoners—1988 to 2002: a data-linkage study. International Journal of Epidemiology 2007; 36: 310–316. 17158524 10.1093/ije/dyl225

[7] Kariminia A, Law MG, Butler TG, Levy MH, Corben SP, Kaldor JM, et al Suicide risk among recently released prisoners in New South Wales. Medical Journal of Australia 2007; 187: 387–390. 17908000 10.5694/j.1326-5377.2007.tb01307.x

[8] Davoli M, Bargagli AM, Perucci CA, Schifano P, Belleudi V, Hickman M, et al Risk of fatal overdose during and after specialist drug treatment, the VEDETTE-study. Addiction 2007; 102(12): 1954–1959. 18031430 10.1111/j.1360-0443.2007.02025.x

[9] Degenhardt L, Randall D, Hall W, Law M, Butler T, Burns L. Mortality among clients of a state-wide opioid pharmacotherapy program over 20 years: Risk factors and lives saved. Drug and Alcohol Dependence 2009; 105: 9–15. 19608355 10.1016/j.drugalcdep.2009.05.021

[10] Cornish R, Macleod J, Strang J, Vickerman P, Hickman M. Risk of death during and after opiate substitution treatment in primary care: prospective observational study in UK General Practice Research Database. British Medical Journal 2010; 341: c5475 doi: 10.1136/bmj.c5475 20978062 10.1136/bmj.c5475 PMC2965139

[11] Hunt IM, Kapur N, Webb R, Robinson J, Burns J, Shaw J, et al Suicide in recently discharged psychiatric patients. Psychological Medicine 2009; 39: 443–449. 18507877 10.1017/S0033291708003644

[12] Amin J, Law MG, Bartlett M, Kaldor JM, Dore GJ. Causes of death after diagnosis of hepatitis B or hepatitis C infection: a large community-based linkage study, Lancet 2006; 368: 938–945. 16962883 10.1016/S0140-6736(06)69374-4

[13] McDonald SA Hutchinson SJ, Bird SM, Mills PR, Dillon J, Bloor M, et al A population-based record-linkage study of mortality in hepatitis C-diagnosed persons with or without HIV co-infection in Scotland. Statistical Methods in Medical Research 2009; 18(3): 271–283. 19036907 10.1177/0962280208094690

[14] Merrall ELC, Bird SM, Hutchinson SJ. A record-linkage study of drug-related death and suicide soon after hospital discharge among drug-treatment clients in Scotland, 1996–2006. Addiction 2013; 108: 377–384. 22925008 10.1111/j.1360-0443.2012.04066.x

[15] McDonald SA, Hutchinson SJ, Mills PR, Bird SM, Robertson C, Dillon JF, et al Diagnosis of hepatitis C virus infection in Scotland's injecting drug user population. Epidemiology & Infection 2010; 138: 393–402. 19723361 10.1017/S0950268809990616

[16] Merrall ELC. *Applications of Statistical Methods to understand Public Health Issues that interface with Criminal Justice*. University of Cambridge PhD Thesis: 2012.

[17] Kendrick S, Clarke J. The Scottish record linkage system. Health Bulletin (Edinburgh) 1993; 51:72–79. 8514493

[18] Jackson GWL. *Drug-related deaths in Scotland in 2000* Occasional Paper No. 5. Edinburgh: General Register Office for Scotland, 2001 Available: http://www.gro-scotland.gov.uk/files/00dd-rep.pdf. Accessed 10 June 2010.

[19] Information Services Division. Important changes to how we present our data. Edinburgh: Substance Misuse Team, Information Services Division, 2009. Available: http://www.drugmisuse.isdscotland.org/publications/local/CIS_FAQ.pdf Accessed 2 January 2011.

[20] Cox DR. Regression models and life-tables. Journal of the Royal Statistical Society: Series B 1972; 34:187–202.

[21] R Development Core Team. R: A language and environment for statistical computing. R Foundation for Statistical Computing, Vienna; Austria, 2011.

[22] McAuley A, Best D, Taylor A, Hunter C, Robertson R. From evidence to policy: the Scottish national naloxone programme. Drugs: Education, Prevention & Policy 2012; 19(4): 309–319.

[23] Information Services Division. National Naloxone Programme Scotland—naloxone kits issued in 2012/13 (revision), 27 May 2014. Available: https://isdscotland.scot.nhs.uk/Health-Topics/Drugs-and-Alcohol-Misuse/Publications/2014-05-27/2014-05-27-Naloxone-Summary.pdf?23278445006. Accessed 27 May 2014.

[24] Rome A, Shaw A, Boyle K. *Reducing Drug Users’ Risk of Overdose* Edinburgh: November 2008. Avaialable: http://www.scotland.gov.uk/Publications/2008/10/30132711/0. Accessed 4 December 2010.

[25] Strang J, Bird SM, Parmar MKB. Take-home emergency naloxone to prevent heroin overdose deaths after prison release; rationale and practicalities for the N-ALIVE randomized trial. Journal of Urban Health 2013; 90: 983–996. 23633090 10.1007/s11524-013-9803-1 PMC3795186

